# Prostatic Abscess due to *Ureaplasma parvum* in a Heart Transplant Recipient: Diagnostic Challenges and Clinical Utility of Metagenomics Next-Generation Sequencing (NGS)

**DOI:** 10.1155/crdi/7511507

**Published:** 2025-11-12

**Authors:** Natalia E. Castillo Almeida, Andre C. Kalil, Carlos A. Gomez

**Affiliations:** ^1^Department of Internal Medicine, Division of Infectious Diseases, Beth Israel Deaconess Medical Center, Boston, Massachusetts, USA; ^2^Department of Internal Medicine, Division of Infectious Diseases, University of Nebraska Medical Center, Omaha, Nebraska, USA

**Keywords:** heart transplantation, metagenomics, *Mycoplasma–Ureaplasma* PCR, prostatic abscess, solid organ transplant, *Ureaplasma*

## Abstract

*Ureaplasma* spp. are small and fastidious bacteria that may cause urogenital infections in healthy adults and, in rare cases, invasive disease. These bacteria have been increasingly recognized in immunocompromised patients and have been associated with hyperammonemia syndrome, particularly in lung transplant recipients. In this context, we present a unique clinical case of *Ureaplasma parvum* prostate abscess, a condition rarely observed in heart transplant recipients, diagnosed using next-generation sequencing (NGS).

## 1. Introduction

*Ureaplasma parvum* and *Ureaplasma urealyticum* are the only two members of the *Ureaplasma* genus. Along with *Mycoplasma genitalium* and *Mycoplasma hominis*, they form the group clinically known as “genital mycoplasmas.” These sexually transmitted pathogens are known to cause urogenital infections in healthy adults and, in rare cases, invasive disease [1, 2]. *Ureaplasmas* have been increasingly recognized in immunocompromised patients, particularly causing hyperammonemia syndrome in lung transplant recipients [3–5]. Conversely, pyogenic urogenital infections are seldom reported. Because *Ureaplasmas* are small and fastidious bacteria incapable of growing in conventional culture media, their diagnosis relies on high clinical suspicion, the use of specialized culture media, and molecular techniques such as targeted polymerase chain reaction (PCR). We present a unique case of *Ureaplasma parvum* prostatic abscess, a condition rarely observed in heart transplant recipients, in which the etiological diagnosis was expedited by metagenomics next-generation sequencing (NGS).

## 2. Case Presentation

**Figure 1:**
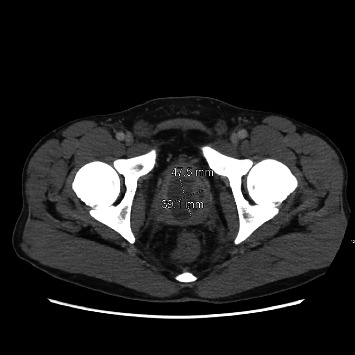
Rim-enhancing multiloculated fluid collection in the prostate measuring 4.8 × 3.8 cm.

A 29-year-old African American patient with a history of heart transplantation at one month of age due to neonatal viral myocarditis was admitted to the hospital due to severe rectal and perineal pain. The patient had been in his usual state of health until 3 weeks before the admission when he started experiencing progressive rectal discomfort, dysuria, and perineal pain that prevented him from sitting or lying comfortably. A week prior, he was diagnosed with an anal fissure with thrombosed hemorrhoids at the emergency department (ED) and discharged with conservative management and steroid suppositories. However, his pain worsened, requiring hospital admission. His immunosuppression consisted of cyclosporine and prednisone, and there was no recent history of allograft rejection. His past medical history included postlymphoproliferative disorder (PTLD), diagnosed 2 years prior, and marginal zone lymphoma, for which he received rituximab and chemotherapy, leading to remission 6 months before the current admission. He reported to be on a monogamous heterosexual relationship and had no history of anal intercourse. Physical examination revealed external hemorrhoids, an anal fissure, and an uncircumcised phallus with significant phimosis that impeded glans exposure. Testicles were descended without tenderness to palpation bilaterally. A rectal exam was not performed. Laboratory tests, including complete blood count, chemistry, and urinalysis, were all within normal limits. Inflammatory markers were mildly elevated (ESR 41, range 0–15 mm/hr; C-reactive protein: 1.39 mg/dL; cutoff < 1.0 mg/dL). Blood and urine cultures were collected shortly after ED arrival and were negative. A contrasted computed tomography of the abdomen and pelvis showed a rim-enhancing multiloculated fluid collection in the prostate (Figure 1). Following hospital admission, he was started on IV vancomycin and piperacillin/tazobactam and underwent cystoscopy and transurethral resection of the prostate. During the procedure, a copious amount of purulent material was noted from the abscess cavity. Surgical specimens were only sent for histopathology evaluation. Urine nucleic acid–based testing (NAT) for *Chlamydia* and *gonorrhea* and syphilis testing were also negative. Considering the pyogenic nature of this infection, a fastidious pathogen was suspected since urine and blood culture specimens were collected before the initiation of broad-spectrum antibiotics. Consequently, advanced molecular testing was pursued, including a plasma microbial cell-free DNA NGS assay-Karius test (KT), urogenital *Ureaplasma*/*Mycoplasma* PCR (urine), *Mycoplasma/Ureaplasma* culture on urea-containing agar (urine), and broad-range PCR (16s ribosomal DNA sequencing) from resected prostatic tissue biopsy. Two days later, the KT returned positive for *Ureaplasma parvum.* Urine culture and targeted PCR in prostate tissue confirmed the presence of *Ureaplasma parvum*. The patient was treated with oral levofloxacin for 6 weeks based on susceptibility testing, and he reported resolution of symptoms upon clinic follow-up 8 weeks later.

## 3. Discussion

The isolation of *Ureaplasma* from urogenital samples is challenging because these organisms are fastidious and require specialized culture media, which are not commonly used in routine microbiologic testing. Additionally, routine *Ureaplasma* testing for uncomplicated genital tract disease is not standard because it is often unclear whether results indicate normal colonization or infection [6]. However, the presence of genitourinary *Ureaplasma* may be higher in immunocompromised individuals, and both cellular and humoral factors may contribute to disseminated and extragenital diseases as highlighted in our case [3, 7, 8]. Furthermore, intracellular localization may contribute to the ability to evade the host immune response. Most cases of invasive *Ureaplasma* disease have been reported in individuals with humoral immunodeficiency, whether it is congenital (hypogammaglobulinemia) or iatrogenic (monoclonal antibody therapy targeted against CD20) [4].

Due to limitations of conventional culture methods, specialized cultures or targeted PCR testing is necessary when there is a clinical suspicion. Real-time PCR has a sensitivity of 96.9% and a specificity of 79% compared to culture [9]. However, there are no FDA-cleared nucleic acid–based assays for these organisms, and different reference laboratories have developed and validated their assays using different gene targets and techniques. Specimen contamination by *Ureaplasma* DNA can occur due to the high sensitivity of PCR amplification. False-negative results can also occur due to PCR inhibition and sequence variability underlying the primers and probes [10].

Due to the limited availability and longer processing time of *Ureaplasma* PCR testing, KT is an emerging infectious disease diagnostic tool that holds promise in providing a timely diagnosis of pathogens that are fastidious to grow using conventional methods or when culture recovery is affected by previous antimicrobial therapy. A recent study evaluated the real-world usefulness of KT in the diagnosis and clinical management of patients with osteoarticular infections. The study yielded encouraging results, with KT enhancing diagnostic accuracy by 11.6%, particularly in diagnosing native vertebral osteomyelitis [11]. The utility of KT in immunocompromised patients has also been explored, albeit in small cohorts or case reports [12, 13]. Given its limited evidence, the complementary role of KT to conventional microbiological methods and its integration into current testing algorithms is still being determined. These limitations include the inability to differentiate commensal versus pathogenic organisms and limited antimicrobial susceptibility data [14].

**Figure 2:**
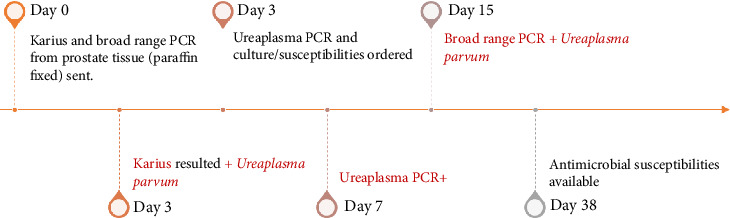
Timeline to diagnose *Ureaplasma parvum* as the culprit for prostatic abscess.

The treatment of *Ureaplasma* infections is often empiric as evidence on susceptibility data is limited [15]. Thus, in patients with severe illness due to suspected or confirmed *Ureaplasma* spp. infection, two different classes of antimicrobials can be used as empiric or targeted therapies. In our patient, the diagnosis of *Ureaplasma* infection was expedited by 4 days using KT upfront (Figure 2), and transmission and reinfection were also minimized by treating his partner. This would have been missed if an isolated pathogen had not been identified and the patient had been treated empirically for acute bacterial prostatitis where antimicrobial coverage focuses primarily on enteric Gram-negative pathogens.

## 4. Conclusion

Further studies are needed to investigate the role of NGS within routine clinical care when facing infectious syndromes that might be missed by conventional testing. The value of incorporating NGS testing into routine clinical practice for high-risk immunocompromised patients should be evaluated prospectively by studies that assess its impact on metrics related to diagnostic accuracy, antimicrobial use, cost savings associated with expedited diagnosis, such as decreasing utilization of further diagnostics and invasive procedures, and particularly maximizing infection prevention and public health.

## Data Availability

Data sharing is not applicable as no new data were generated.

## Nomenclature

PCR: Polymerase chain reaction
NGS: Next-generation sequencing
PTLD: Postlymphoproliferative disorder
ED: Emergency department
NAT: Nucleic acid–based testing
KT: Karius testing
